# The PTEN tumor suppressor gene and its role in lymphoma pathogenesis

**DOI:** 10.18632/aging.100855

**Published:** 2015-12-10

**Authors:** Xiaoxiao Wang, Huiqiang Huang, Ken H. Young

**Affiliations:** ^1^ Department of Hematopathology, The University of Texas M. D. Anderson Cancer Center, Houston, TX 77230, USA; ^2^ Department of Medical Oncology, Sun Yat-Sen University Cancer Center, Guangzhou, Guangdong, China; ^3^ The University of Texas Graduate School of Biomedical Science, Houston, TX 77230, USA

**Keywords:** PTEN, tumor suppressor, PI3K, AKT, mTOR, lymphoid malignancies, diffuse large B-cell lymphoma

## Abstract

The phosphatase and tensin homolog gene *PTEN* is one of the most frequently mutated tumor suppressor genes in human cancer. Loss of PTEN function occurs in a variety of human cancers via its mutation, deletion, transcriptional silencing, or protein instability. PTEN deficiency in cancer has been associated with advanced disease, chemotherapy resistance, and poor survival. Impaired PTEN function, which antagonizes phosphoinositide 3-kinase (PI3K) signaling, causes the accumulation of phosphatidylinositol (3,4,5)-triphosphate and thereby the suppression of downstream components of the PI3K pathway, including the protein kinase B and mammalian target of rapamycin kinases. In addition to having lipid phosphorylation activity, PTEN has critical roles in the regulation of genomic instability, DNA repair, stem cell self-renewal, cellular senescence, and cell migration. Although PTEN deficiency in solid tumors has been studied extensively, rare studies have investigated PTEN alteration in lymphoid malignancies. However, genomic or epigenomic aberrations of *PTEN* and dysregulated signaling are likely critical in lymphoma pathogenesis and progression. This review provides updated summary on the role of PTEN deficiency in human cancers, specifically in lymphoid malignancies; the molecular mechanisms of PTEN regulation; and the distinct functions of nuclear PTEN. Therapeutic strategies for rescuing PTEN deficiency in human cancers are proposed.

## INTRODUCTION

The phosphatase and tensin homolog gene,* PTEN*, is one of the most commonly mutated tumor suppressors in human malignancies [1–5], and complete loss of PTEN protein expression is significantly associated with advanced cancer and poor outcome [6, 7]. The importance of *PTEN* as a tumor suppressor is further supported by the fact that germline mutations of *PTEN* commonly occur in a group of autosomal dominant syndromes, including Cowden Syndrome, which are characterized by developmental disorders, neurological deficits, and an increased lifetime risk of cancer and are collectively referred to as PTEN hamartoma tumor syndromes (PHTS) [8, 9].

Biochemically, PTEN is a phosphatase that de-phosphorylates phosphatidylinositol (3,4,5)-tri-phosphate (PIP_3_), the lipid product of class I phosphoinositide 3-kinase (PI3K) [10]. To date, PTEN is the only lipid phosphatase known to counteract the PI3K pathway. Unsurprisingly, loss of PTEN has a substantial impact on multiple aspects of cancer development. Strikingly, PTEN has distinct growth-regulatory roles depending on whether it is in the cytoplasm or nucleus. In the cytoplasm, PTEN has intrinsic lipid phosphatase activity that negatively regulates the cytoplasmic PI3K/AKT pathway, whereas in the nucleus, PTEN has AKT-independent growth activities. The continued elucidation of the roles of nuclear PTEN will help uncover the various functions of this essential tumor suppressor gene.

In this review, we describe the molecular basis of PTEN loss, discuss the regulation of PTEN expression in lymphoid malignancies, and summarize potential therapeutic targets in PTEN-deficient cancers.

## STRUCTURE AND FUNCTION OF PTEN

### PTEN structure

**Figure 1:**
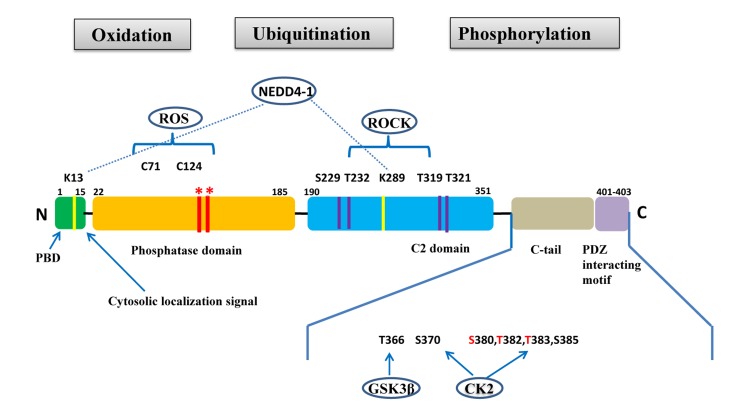
PTEN gene and protein structures The PTEN protein is composed of 403 amino acids and contains an N-terminal PIP_2_-binding domain (PBD), a phosphatase domain, a C2 domain, a C-terminal tail containing proline–glutamic acid–serine–threonine sequences, and a PDZ interacting motif at the end. *Mutations on the phosphatase domain that disrupt PTEN's phosphatase activity include the C124S mutation, which abrogates both the lipid and protein phosphatase activity of PTEN, and the G129E mutation, which abrogates only the lipid phosphatase activity of PTEN. The C-terminal tail residues phosphorylated by glycogen synthase kinase 3β (GSK3β) and casein kinase 2 (CK2) are shown. Mutations of S380, T382, and T383 (referred to as the STT) can destabilize PTEN and increase its phosphatase activity. The PIP_2_-binding site and adjacent cytoplasmic localization signal are located at the N-terminal. The N-terminal poly-basic region appears to selectively interact with PIP_2_ and contribute to the nuclear accumulation of PTEN. Ubiquitination of PTEN has also been found on K13 and K289.

*PTEN* is a tumor suppressor gene located on chromosome 10q23.31 that encodes for a 403-amino acid protein that has both lipid and protein phosphatase activities. PTEN gene and protein structures are shown in Figure 1. The PTEN protein contains a sequence motif that is highly conserved in members of the protein tyrosine phosphatase family. Structurally, the PTEN protein is composed of two major functional domains (a phosphatase domain and a C2 domain) and three structural regions (a short N-terminal phosphatidyl-inositol [4,5]-bisphosphate [PIP_2_]-binding domain, a C-terminal tail containing proline-glutamic acid-serine-threonine sequences, and a PDZ-interaction motif) [11]. The PIP_2_-binding site and adjacent cytoplasmic localization signal are located at the protein's N-terminal [12, 13].

### The PI3K/PTEN/AKT/mTOR pathway

PTEN's tumor-suppressing function largely relies on the protein's phosphatase activity and subsequent antagonism of the PI3K/AKT/mammalian target of rapamycin (mTOR) pathway. Following PTEN loss, excessive PIP_3_ at the plasma membrane recruits and activates a subset of pleckstrin homology domain–containing proteins to the cell membrane. These proteins include phosphoinositide-dependent kinase-1 and AKT family members [14, 15]. AKT activation also leads to the activation of the mTOR kinase complex 1 through the inhibition of the phosphorylation of tuberous sclerosis complex tumor suppressors and consequent activation of the small GTPase rat sarcoma (RAS) homologue enriched in brain. The active mTOR complex 1 phosphorylates the p70 ribosomal protein S6 kinase (S6K) and inhibits 4E-binding protein 1 to activate protein translation [16]. Accordingly, the PTEN/PI3K/AKT/mTOR pathway is emerging as a vital target for anti-cancer agents, especially in tumors with mTOR pathway activation.

### AKT-independent roles of PTEN

Although AKT pathway activation can explain many of the phenotypes associated with *PTEN* inactivation, PTEN gene targeting and genetic activation of *AKT* do not have completely overlapping biological consequences. Using transcriptional profiling, Vivanco et al. identified a new PTEN-regulated pathway, the Jun-N-terminal kinase (JNK) pathway, which was constitutively activated upon PTEN knockdown [17]. In the study, PTEN null cells had higher JNK activity than PTEN positive cells did, and genetic analysis indicated that JNK functioned parallel to and independently of AKT. Thus, the blockade of PI3K signaling may shift the survival signal to the AKT-independent PTEN-regulated pathway, implicated JNK and AKT as complementary signals in PIP_3_-driven tumorigenesis and suggest that JNK may be a therapeutic target in *PTEN* null tumors.

In addition to its lipid phosphatase function, PTEN also has lipid phosphatase–independent roles. PTEN has been shown to inhibit cell migration through its C2 domain, independent of PTEN's lipid phosphatase activity [18]. In breast cancer, PTEN deficiency has been shown to activate, in a manner dependent on its protein phosphatase activity, the SRC proto-oncogene, non-receptor tyrosine kinase (SRC), thereby conferring resistance to human epidermal growth factor receptor 2 inhibition [19]. Furthermore, PTEN has been shown to directly bind to tumor protein 53 (p53), regulate its stability, and increase its transcription, thereby increasing P53 protein levels [20].

## PTEN REGULATION

### Genetic alteration of *PTEN*

PTEN loss of function occurs in a wide spectrum of human cancers through various genetic alterations that include point mutations (missense and nonsense mutations), large chromosomal deletions (homozygous/heterozygous deletions, frameshift deletions, in-frame deletions, and truncations), and epigenetic mechanisms (e.g., hypermethylation of the *PTEN* promoter region) [21]. Somatic mutations are the main drivers of PTEN inactivation in human cancers, and have been reviewed extensively [22].

PTEN's tumor suppressor function is usually abrogated following mutations in its phosphatase domain, which is encoded by exon 5 [23] (Figure 1). These mutations typically include a C124S mutation that abrogates both lipid and protein phosphatase activity and a G129E mutation that abrogates lipid phosphatase but not protein phosphatase activity [24]. Although the N-terminal phosphatase domain is principally responsible for PTEN's physiological activity, approximately 40% of tumorigenic *PTEN* mutations occur in the C-terminal C2 domain (corresponding to exons 6, 7, and 8) and in the tail sequence (corresponding to exon 9), which encode for tyrosine kinase phosphorylation sites. This suggests that the C-terminal sequence is critical for maintaining PTEN function and protein stability [21, 23, 25, 26]. However, many tumor-derived *PTEN* mutants retain partial or complete catalytic function, suggesting that alterative mechanisms can lead to PTEN inactivation.

### Transcriptional regulation

**Figure 2:**
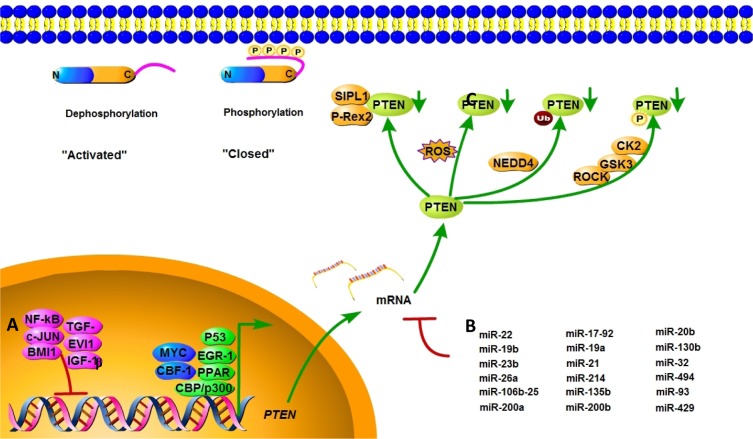
Mechanisms of PTEN regulation PTEN is regulated at different levels. (**A**) PTEN mRNA transcription is activated by early growth response protein 1, P53, MYC, PPARγ, C-repeat binding factor 1, and others, and inhibited by NF-κB, proto-oncogene c-Jun, TGF-β, and BMI-1. (**B**) PTEN mRNA is also post-transcriptionally regulated by PTEN-targeting miRNAs, including miR-21, miR-17-92, and others. (**C**) Active site phosphorylation, ubiquitination, oxidation, acetylation, and protein-protein interactions can also regulate PTEN activity. The phosphorylation leads to a “closed” state of PTEN and maintains PTEN stability. Dephosphorylation of the C-terminal tail opens the PTEN phosphatase domain, thereby activating PTEN.

In addition to gene mutations, complete or partial loss of PTEN protein expression may impact PTEN's tumor suppression ability. The regulation of PTEN's functions and signaling pathway is shown in Figure 2. Positive regulators of PTEN gene expression include early growth response protein 1, peroxisome proliferator-activated receptor γ (PPARγ) and P53, which have been shown to directly bind to the PTEN promoter region [27–29]. Early growth response protein 1, which regulates PTEN expression during the initial steps of apoptosis, has been shown to directly upregulate the expression of PTEN in non–small cell lung cancer. PPARγ is a ligand-activated transcription factor with anti-inflammatory and anti-tumor effects. The activation of its selective ligand, rosiglitazone, leads to the binding of PPARγ at two PTEN promoter sites, PPAR response element 1 and PPAR response element 2, thus upregulating PTEN and inhibiting PI3K activity. Negative regulators of PTEN gene expression include mitogen-activated protein kinase kinase-4, transforming growth factor beta (TGF-β), nuclear factor of kappa light polypeptide gene enhancer in B-cells (NF-κB), IGF-1, the transcriptional cofactor c-Jun proto-oncogene, and the B-cell-specific Moloney murine leukemia virus insertion site 1 (BMI1) proto-oncogene, which have been shown to suppress PTEN expression in several cancer models [30–32]. Research found that IGF-1 could affect cell proliferation and invasion by suppressing PTEN's phosphorylation. In pancreatic cancers, TGF-β significantly suppresses PTEN protein levels concomitant with the activation of AKT through transcriptional reduction of PTEN mRNA–induced growth promotion. c-Jun negatively regulates the expression of PTEN by binding to the activator protein 1 site of the PTEN promoter, resulting in the concomitant activation of the AKT pathway. *PTEN* transcription is also directly repressed by the leukemia-associated factor ecotropic virus integration site 1 protein in the hematopoietic system [33].

Intriguingly, recent studies reported a complex crosstalk between PTEN and other pathways. For example, RAS has been found to mediate the suppression of PTEN through a TGF-β dependent mechanism in pancreatic cancer [34], and the mitogen-associated protein kinase/extracellular signal-related kinase pathway has been found to suppress PTEN transcription through c-Jun [35]. Finally, the stress kinase pathways including mitogen-activated protein kinase kinase kinase 4 and JNK promote resistance to apoptosis by suppressing PTEN transcription via direct binding of NF-κB to the PTEN promoter [36]. These findings suggest that the pathways that are negatively regulated by PTEN can in turn regulate PTEN transcription, indicating a potential feedback loop. Studies have also shown that CpG islands hypermethylated in the *PTEN* promoter lead to the silencing of *PTEN* transcription in human cancer [37].

### Translational and post-translational regulation

MicroRNAs (miRNAs) are a class of endogenous, 20- to 25-nucleotide single-stranded non-coding RNAs that repress mRNA translation by base-pairing with target mRNAs [38]. Various miRNAs are known to impact PTEN expression in both normal and pathological conditions. In multiple human cancers, PTEN expressions are downregulated by miRNAs, which are shown in Table 1.

**Table 1:** MiRNAs which downregulate PTEN expression in human cancers

miRNA	Locus	Expression status	Tumor type	Reference
MiR-21	17q23.1	Upregulated	Colorectal, bladder, and hepatocellular cancer	[112–114]
MiR-19a	13q31.3	Upregulated	Lymphoma and CLL	[87, 115]
MiR-19b	Xq26.2	Upregulated	Lymphoma	[87]
MiR-22	17p13.3	Upregulated	Prostate cancer and CLL	[116, 117]
MiR-32	9q31.3	Upregulated	Hepatocellular carcinoma	[118]
MiR-93	7q22.1	Upregulated	Hepatocellular carcinoma	[119]
MiR-494	14q32.31	Upregulated	Cervical cancer	[120]
MiR-130b	22q11.21	Upregulated	Esophageal carcinoma	[121]
MiR-135b	1q32.1	Upregulated	Colorectal cancer	[122]
MiR-214	1q24.3	Upregulated	Ovarian cancer	[123]
MiR-26a	3p22.2 (MIR26A1) 12q14.1(MIR26A2)	Upregulated	Prostate cancer	[113]
MiR-23b	9q22.32	Upregulated	Prostate cancer	[114]

Abbreviations: CLL, chronic lymphocytic leukemia.

**Figure 3:**
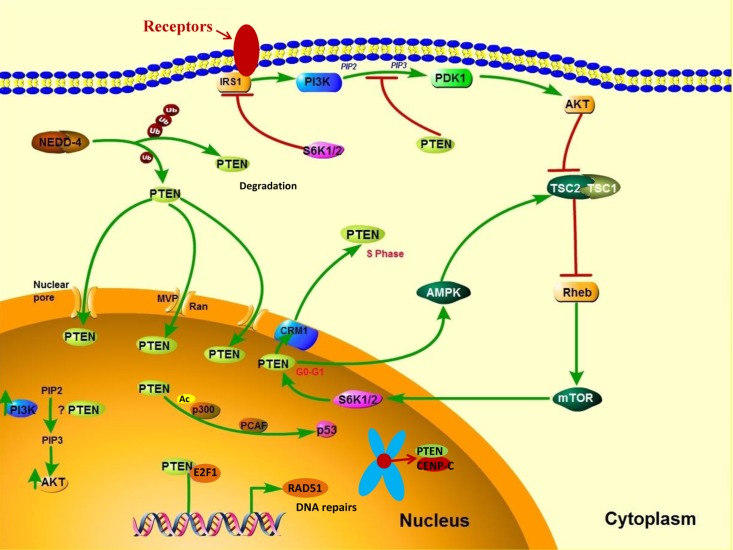
PTEN's cytoplasmic and nuclear functions In the cytoplasm, PI3K is activated downstream of receptors that include receptor tyrosine kinases, G protein–coupled receptors, cytokine receptors, and integrins. PI3K activation converts PIP_2_ to PIP_3_, thereby leading to AKT activation, which enhances cell growth, proliferation, and survival. PTEN dephosphorylates PIP_3_ and consequently suppresses the PI3K pathway. NEDD4-1 is an E3 ligase of PTEN that mediates PTEN ubiquitination. Polyubiquitination of PTEN leads to its degradation in the plasma, whereas monoubiquitination of PTEN increases its nuclear localization. PTEN can translocate into the nucleus through various mechanisms, including passive diffusion, Ran- or major vault protein–mediated import, and a monoubiquitination-driven mechanism. In the nucleus, PTEN promotes p300-mediated P53 acetylation in response to DNA damage to control cellular proliferation. Nuclear PTEN is also involved in maintaining genomic integrity by binding to centromere protein C (CENPC) and in DNA repair by upregulating RAD51 recombinase (RAD51).

Post-translational modifications, such as active site phosphorylation, ubiquitination, oxidation, and acetylation, can also regulate PTEN activity [39]. In its inactivated state, PTEN is phosphorylated on a cluster of serine and threonine residues located on its C-terminal tail, leading to a “closed” PTEN state in which PTEN protein stability is maintained. As PTEN is being activated, dephosphorylation of its C-terminal tail opens its phosphatase domain, thereby increasing PTEN activity (Figure 2). The phosphorylation of PTEN at specific residues of the C-terminal tail (Thr366, Ser370, Ser380, Thr382, Thr383, and Ser385) is associated with increased protein stability, whereas phosphorylation at other sites may decrease protein stability. Although S370 and S385 have been identified as the major sites for PTEN phosphorylation, mutations of these residues have minimal effects on PTEN function, whereas mutations of S380, T382, and T383 can destabilize PTEN and increase its phosphatase activity, thereby enhancing PTEN's interaction with binding partners [40]. The “open” state of PTEN is more susceptible to ubiquitin-mediated proteasome degradation [13]. One recently identified E3 ligase of PTEN is neural precursor cell-expressed, developmentally down-regulated 4, E3 ubiquitin protein ligase 1 (NEDD4-1), which mediates PTEN mono- and poly-ubiquitination [41] (Figure 3). In cancer, the inhibition of NEDD4-1, whose expression has been found to be inversely correlated with PTEN levels in bladder cancer, may upregulate PTEN levels [42]. Two major conserved sites for PTEN are K13 and K289, and ubiquitination of these sites is indispensable for the nuclear-cytoplasmic shuttling of PTEN (Figure 1).

### Protein-protein interactions

PTEN contains a 3–amino acid C-terminal region that is able to bind to PDZ domain–containing proteins [43, 44]. PDZ domains are involved in the assembly of multi-protein complexes that may control the localization of PTEN and its interaction with other proteins. A number of PTEN-interacting proteins have been shown to regulate PTEN protein levels and activities. These interactions, which help recruit PTEN to the membrane, can be negatively modulated by the phosphorylation of PTEN on its C terminus [40, 45]. The phosphorylation of the C terminal end of PTEN has been attributed to the activities of casein kinase 2 and glycogen synthase kinase 3β [46, 47]. In addition, evidence suggests that the C2 domain of PTEN can be phosphorylated by RhoA-associated kinase, which may have important roles in the regulation of chemoattractant-induced PTEN localization [48] (Figure 2).

Acetylation and oxidation also contribute to PTEN activity regulation. PTEN's interaction with nuclear histone acetyltransferase–associated p300/cAMP response element-binding protein (CREB)-binding protein (CBP)–associated factor can promote PTEN acetylation, and this acetylation negatively regulates the catalytic activity of PTEN [49]. Studies have shown that the PTEN protein becomes oxidized in response to the endogenous generation of the reactive oxygen species (ROS) stimulated by growth factors and insulin, and this oxidation correlates with a ROS-dependent activation of downstream AKT phosphorylation [50, 51]. Other studies have shown that the PIP_3_-dependent Rac exchange factor 2 and SHANK-associated RH domain interactor proteins bind directly to PTEN to inhibit its lipid phosphatase activity [52, 53]. High P53 expression triggers proteasome degradation of the PTEN protein [54]. In addition to antagonizing the AKT–mouse double minute 2 homolog pathway in a phosphatase-dependent manner, PTEN also can interact with P53 directly in a phosphatase-independent manner, thereby stabilizing P53 [55, 56].

## PTEN IN THE NUCLEUS

Growing evidence suggests that the translocation of PTEN from the nucleus to the cytoplasm leads to malignancy. In the nucleus, PTEN has important tumor-suppressive functions, and the absence of nuclear PTEN is associated with aggressive disease in multiple cancers [57–59], implying that nuclear PTEN is a useful prognostic indicator. PTEN is predominantly localized to the nucleus in primary, differentiated, and resting cells, and nuclear PTEN is markedly reduced in rapidly cycling cancer cells [60, 61], which suggests that PTEN localization is related to cell differentiation status and cell cycle stage. High expression levels of nuclear PTEN have been associated with cell-cycle arrest at the G0/G1 phase, indicating a role of nuclear PTEN in cell growth inhibition [62]. PTEN's cytoplasmic and nuclear functions are shown in Figure 3.

PTEN enters the nucleus via its calcium-dependent interaction with the major vault protein [63], through passive diffusion [64], and by a Ran-GTPase–dependent pathway [65]. Moreover, monoubiquitination mediates PTEN's nuclear import, whereas polyubiquitination leads to PTEN's degradation in the cytoplasm [66] (Figure 3). The nuclear exportation of PTEN via a chromosome region maintenance 1–dependent mechanism during the G1-S phase transition is directly regulated by S6K, a downstream effector of the PI3K signaling pathway [67] (Figure 3). Thus, PTEN is preferentially expressed in the cytoplasm of tumor cells in which PI3K signaling is frequently activated. Nuclear PTEN has an essential role in the maintenance of chromosomal stability. First, PTEN directly interacts with centromere protein C in a phosphatase-independent manner. Second, PTEN transcriptionally regulates DNA repair by upregulating RAD51 recombinase in a phosphatase-dependent manner [68] (Figure 3). The disruption of nuclear PTEN results in centromere breakage and massive chromosomal aberrations. Nuclear PTEN may also play an important part in transcription regulation by negatively modulating the transcriptional activity of the androgen receptor, hepatocyte growth factor receptor, NF-κB, CREB, and activator protein 1. Moreover, nuclear PTEN has been shown to promote p300/CREB-binding protein–mediated p53 acetylation in the response to DNA damage [69, 70].

Most of the functions of nuclear PTEN are independent of its phosphatase activity and do not involve the PI3K/AKT pathway. Not only PTEN but also activated PI3K and functional PIP_3_ have been detected in the nucleus [71], indicating that nuclear PI3K signaling mediates PTEN's antiapoptotic effect through nuclear PIP_3_ and nuclear AKT. Nevertheless, only limited evidence suggests that nuclear PTEN has lipid phosphatase functions, as the nuclear pool of PIP_3_ is insensitive to PTEN [72].

## PTEN DEFICIENCY IN LYMPHOMA

### PTEN deficiency in T-cell acute lymphoblastic leukemia

PI3K signaling are frequently activated in T-cell acute lymphoblastic leukemia (T-ALL), which mainly due to the absent of PTEN function. Studies have shown that *PTEN* inactivation plays a prominent role in human T-ALL cell lines and primary patients [73–76]. Moreover, *PTEN* mutations have been shown induced resistance to γ-secretase inhibitors, which derepress the constitutively activated NOTCH1 signaling in T-ALL [77]. However, the *PTEN* mutations detected in these studies vary widely. Gutierrez et al. reported that T-ALL patients had a *PTEN* mutation rate of 27% and a *PTEN* deletion rate of 9%, whereas Gedman et al. reported that 27 of 43 (63%) pediatric T-ALL specimens had *PTEN* mutations. In the latter study, the high frequency of *PTEN* mutations may have been due to the fact that approximately 50% of the specimens were patients with relapsed disease. Interestingly, all mutations were identified in the C2 domain of PTEN [75, 76], not in the phosphatase domain as has been reported for other solid tumors [78].

### PTEN deficiency in diffuse large B-cell lymphoma

Published reports of *PTEN* gene alterations in lymphoid malignancies are summarized in Table 2. Studies have reported unexpectedly low frequencies of* PTEN* mutations in DLBCL patients, ranging from 3% to 22% [79–83]. Lenz et al. performed gene expression profiling in primary DLBCL and found that a recurrently altered minimal common region containing *PTEN* was lost in 11% GCB-DLBCL but not in other subtypes, suggesting that the alteration is exclusive to GCB-DLBCL [84]. More recently, Pfeifer and Lenz found that mutations involving both the phosphatase domain and C2 domain of *PTEN* were prominent in GCB-DLBCL cell lines. Interestingly, 7 of the 11 GCB-DLBCL cell lines had complete loss of PTEN function, whereas all ABC-DLBCL cell lines expressed PTEN, suggesting that *PTEN* mutation may be related to PTEN loss in GCB-DLBCL [85] (Table 2). In the GCB-DLBCL cell lines, PTEN loss was inversely correlated with the constitutive activation of the PI3K/AKT signaling pathway, whereas GCB-DLBCL cell lines with PTEN expression rarely had PI3K/AKT activation. In contrast, all ABC-DLBCL cell lines had PI3K/AKT activation regardless of PTEN status, which suggests that the activation of PI3K/AKT in GCB-DLBCL results from PTEN deficiency. Further, gene set enrichment analysis revealed that the MYC target gene set was significantly downregulated after PTEN induction. Also, inhibition of PI3K/AKT with either PTEN re-expression or PI3K inhibition significantly downregulated MYC expression, suggesting that PTEN loss leads to the upregulation of MYC through the constitutive activation of PI3K/AKT in DLBCL [85].

**Table 2:** Reported *PTEN* gene alterations in lymphoid malignancies

Alteration type	Exon	Domain	Disease	Frequency, %	Notes	Ref
Cell lines						
Del	3-9	PHOS, C2	DLBCL	28.6 (4/14)	Del in 4 of 11 GCB-DLBCL	[85]
Mut	2-5	PHOS, C2	DLBCL	35.7 (5/14)	Mut in 4 of 11 GCB- and 1 of 3 ABC-DLBCL	
Del and Mut	2-7	PHOS, C2		22.2 (6/27)		[82]
Biopsy tissue						
Del			DLBCL	15.3 (4/26)	Heterozygous Del in 3 of 18 GCB- and 1 of 8 ABC-DLBCL	[85]
Del	1	PB	NHL	3.4 (1/29)		[81]
Mut	5, 6	PHOS, C2		6.9 (2/29)		
Del and Mut	1, 8	PHOS, C2	NHL	4.6 (3/65)		[82]
Mut	8	C2	DLBCL	5 (2/39)		[79]
Del			GCB-DLBCL	13.9 (10/72)	Homozygous Del in 2, heterozygous Del in 8	[84]
Mut	1, 2, 7	PB, PHOS, C2	NHL	10 (4/40)		[109]
Mut	7	C2	T-ALL	8 (9/111)		[74]
Del	NA		T-ALL	8.7 (4/46)	Homozygous Del in 2, heterozygous Del in 2	[76]
Mut	7	C2		27.3 (12/44)		
Del and Mut			T-ALL	62.7 (27/43)	Homozygous Del in 8	[75]

Abbreviations: Del, deletion; PHOS, phosphatase; DLBCL, diffuse large B-cell lymphoma; GCB, germinal B-cell–like; Mut, mutation; ABC, activated B-cell–like; NHL, non-Hodgkin lymphoma; T-ALL, T-cell acute lymphoblastic leukemia; AML, acute myeloid leukemia; ALL, acute lymphoblastic leukemia; PB, phosphatidylinositol (4,5)-bisphosphate–binding; NA, not applicable.

Although several studies have identified discrepancies in PTEN deficiency between DLBCL subtypes, few studies have investigated PTEN localization in different subcellular compartments, not to mention the prognostic value such information would have in de novo cases. Fridberg et al. found a trend towards a stronger staining intensity of cytoplasmic and nuclear PTEN in 28 non–GCB-DLBCL patients [59], most importantly, they found that the absence of nuclear PTEN expression was correlated with worse survival. This interesting evidence should be corroborated in a larger number of primary samples in further studies.

### PTEN deficiency in other lymphomas

Previous studies of mantle cell lymphoma (MCL) showed that although the disease had no detectable genetic alterations of PTEN, it did have extremely low protein expression of PTEN. To determine whether the PI3K/AKT signaling pathway is involved in the pathogenesis of MCL, Rudelius et al. investigated pAKT and PTEN expression in primary MCL specimens and cell lines. Of the 31 MCL specimens, 6 had markedly decreased PTEN expression; of the 4 MCL cell lines, 3 had complete loss of PTEN expression [86]. The authors found no phosphatidyl inositol 3-kinase catalytic subunit (*PIK3CA*) mutations in the primary specimens or cell lines, suggesting that loss of PTEN activates the PI3K/AKT pathway in MCL.

Loss of PTEN protein expression has also been reported in 32% of patients with primary cutaneous DLBCL–leg type and 27% of patients with primary cutaneous follicle center lymphomas. Remarkably, both the expression of miR-106a and that of miR-20a were significantly related to PTEN protein loss (P<0.01). Moreover, low PTEN mRNA levels were significantly associated with shorter disease-free survival [87].

## PTEN AND SPECIFIC PI3K ISOFORMS

PI3K comprises a regulatory p85 subunit and a catalytic p110 subunit. Of particular interest, Class IA PI3Ks include three p110 isoforms (p110α, p110β, and p110δ), are primarily responsible for phosphorylating PIP_2_. *PIK3CA*, the gene encoding the p110α isoform is frequently mutated in various human cancers [88]. In one study, 59% of cases with mutant *PIK3CA* had increased p-AKT levels. Therefore, the constitutive activation of PI3K is another way by which the PTEN pathway can be disturbed in cancer. In their study of 215 DLBCL patients, Abubaker et al. reported that 8% had *PIK3CA* mutations and 37% had loss of PTEN. Both *PIK3CA* mutation and loss of PTEN were correlated with poor survival. However, correlation analysis revealed that most of the *PIK3CA* mutations occurred in cases with PTEN expression (P=0.0146). Accordingly, 17 cases with *PIK3CA* mutations were screened for *PTEN* mutations, and none harbored both *PIK3CA* and *PTEN* mutations [89]. This suggests that *PIK3CA* mutation likely functions as an oncogene in DLBCL by contributing to PI3K pathway activation independently of PTEN deficiency.

Both p110α and p110β may generate distinct pools of PIP_3_. In response to stimuli, p110α produces an acute flux of PIP_3_, which is efficiently coupled to AKT phosphorylation. In contrast, p110β has been proposed to generate a basal level of PIP_3_ with little effect on AKT phosphorylation [90]. Moreover, cells with AKT phosphorylation induced by PTEN loss were sensitive to a p110β-specific inhibitor but not a p110α inhibitor both in vitro and in vivo [91, 92], which suggests that the enhancement of basal PIP_3_ drive oncogenesis in the absence of PTEN. Another study indicated that *PTEN*-mutant endometrioid endometrial carcinoma cells may not be sufficiently sensitive to the inhibition of p110β alone and that combined targeted agents may be required for effective treatment [93]. This finding may have been due to the fact that mutations of *PTEN* and *PIK3CA* frequently coexist in endometrioid endometrial carcinoma. In contrast, cells with wild-type PTEN seem to engage the p110α or p110δ isoforms. Accordingly, clinical trials of isoform-specific inhibitors are warranted.

## ENGAGEMENT OF THE PI3K PATHWAY IN B-CELL RECEPTOR SIGNALING

The survival of the majority of B-cell malignancies depends on functional B-cell receptor (BCR) signaling. The successful use of a Bruton tyrosine kinase (BTK) inhibitor to target the BCR pathway in DLBCL has yielded profound discoveries regarding the genetic and biochemical basis of BCR signaling. During BCR signaling, the SRC family kinase LYN phosphorylates the transmembrane protein cluster of differentiation 19, which recruits PI3K to the BCR. The transduction of BCR signaling finally results in the activation of the NF-κB, PI3K, mitogen-associated protein kinase, and nuclear factor of activated T cells pathways, which promote the proliferation and survival of normal and malignant B cells.

BCR signaling is directly affected by frequent mutations in CD79A (immunoglobulin α) and CD79B (immunoglobulin β)-mainly CD79B-which occur in approximately 20% of patients with ABC-DLBCL [94]. Tumor cells harboring CD79B mutations have longer and stronger activation of AKT signaling. Moreover, ABC-DLBCL cell lines with mutated CD79B are more sensitive to PI3K inhibition than those with wild-type CD79B are. Thus, CD79B mutations might be responsible for preventing the negative regulation that interferes with PI3K-dependent pro-survival BCR signaling [95].

Previous studies have demonstrated that the transgenic expression of the constitutively active form of the PI3K catalytic subunit or PTEN knockout can rescue mature B cells from conditional BCR ablation. Moreover, BCR signaling is required for PI3K pathway engagement in both GCB-DLBCL and ABC-DLBCL. Specifically, PI3K engages BCR signaling by indirectly contributing to NF-κB activity in ABC-DLBCL, whereas in GCB-DLBCL, PI3K pathway activation but not NF-κB activity is required for survival. Briefly, the “chronic” BCR signaling in ABC-DLBCL is characterized by the many pathways involved with the CARD11-mediated activation of NF-κB signaling, whereas the “tonic” BCR signaling in GCB-DLBCL is characterized by the constitutive activation of PI3K in promoting survival [96, 97].

Given these findings, the combination of PI3K pathway inhibitors with BCR pathway inhibitors may enhance the treatment response of PTEN-deficient tumors.

## THERAPIES TARGETING FUNCTIONAL LOSS OF PTEN IN LYMPHOMA

### PI3K/AKT/mTOR pathway inhibitors

**Figure 4:**
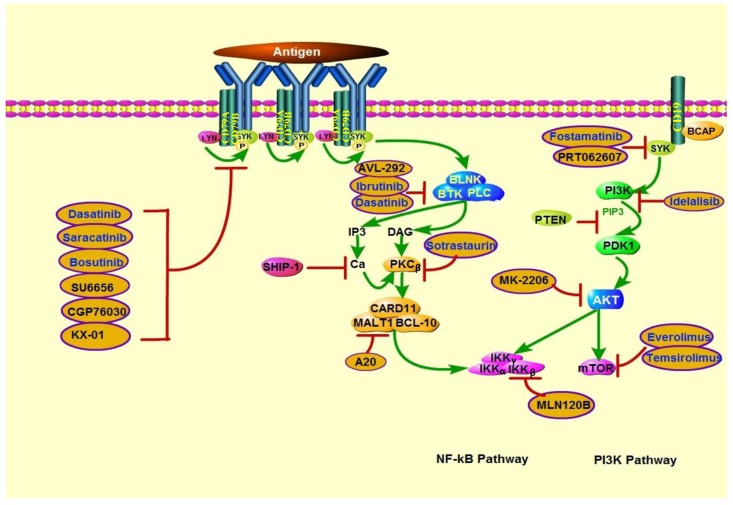
Actions of therapeutics targeting PTEN deficiency in lymphoid malignancies PTEN deficiency is associated with increased sensitivity to PI3K, AKT, and mTOR inhibitors. In addition, because PI3K is involved in BCR signaling activation, BCR pathway inhibitors may also be effective in PTEN-deficient lymphoid malignancies. SRC family kinase inhibitors include dasatinib (which can also inhibit BTK), saracatinib, bosutinib, SU6656, CGP76030, and KX-01. BTK inhibitors include ibrutinib and AVL-292. Sotrastaurin is a PKCβ inhibitor; A20, a MALT1 paracaspase inhibitor; and MLN120B, an IKKβ inhibitor. SYK inhibitors include fostamatinib and PRT062607. Idelalisib is a PI3Kδ-specific inhibitor. MK-2206 is an AKT inhibitor. mTOR inhibitors include everolimus and temsirolimus.

Owing to PI3K's critical roles in human cancers, PI3K targeting is one of the most promising areas of anticancer therapy development. Since the absent of PTEN is concomitant with PI3K signaling activation, inhibitors that targeting this pathway might play a significant role in the treatment of PTEN-deficient tumors. Growing evidence indicates that multiple solid tumor cell lines and several lymphoid malignancy cell lines with PTEN-deficient are hypersensitive to PI3K inhibitors, which are summarized in Tables 3 and Figure 4.

**Table 3:** Preclinical studies of targeted therapeutics in PTEN-deficient tumors

Inhibitor type	Drug	Study notes	Ref
*Class I-PI3K *			
Pan	Buparlisib (BKM120)	The drug elicited response in some PTEN-deficient tumors and induced cell death in DLBCL cell lines.	[124]
Pan	SAR245408 (XL147)	The drug significantly inhibited tumor growth in a PTEN-deficient prostate cancer model.	[109]
p110α	BYL719	The drug had antitumor activity in cell lines harboring *PIK3CA *mutations but not in PTEN-deficient solid tumors	[110]
p110β	AZD6482 (KIN-193)	The drug substantially inhibited tumor growth in PTEN-deficient cancer models.	[98]
p110β	GSK2636771	*PTEN*-mutant EEC cell lines were resistant to the drug; the drug decreased cell viability only when combined with a p110α selective inhibitor.	[93]
p110β/δ	AZD8186	The drug inhibited the growth of PTEN-deficient prostate tumors.	[102]
p110α/β	CH5132799	The drug inhibited the growth of some PTEN-deficient tumors in vitro.	[103]
p110γ/δ	IPI-145	The drug significantly inhibited the Loucy cell lines in T-ALL.	[100]
*PI3K/mTOR*	SF1126	The drug significantly reduced the viability of PTEN-deficient but not PTEN-positive GCB-DLBCL cells.	[104]
*PI3K/HDAC *	CUDC-907	The drug inhibited growth in multiple cell lines; cell lines with *PIK3CA *or *PTEN*-mutation induced loss of PTEN were markedly sensitive to the drug.	[105]
*AKT*	MK-2206	The drug had antitumor activity in breast cancer cell lines with *PTEN *or *PIK3CA *mutations.	[106]
*mTORC1 *	Everolimus (RAD001)	PTEN-deficient prostate cancer had greater sensitivity to the drug; glioblastoma cell lines were resistant to the drug.	[107]
	Temsirolimus (CCI-779)	Multiple PTEN-deficient cell lines were remarkably sensitive to the drug.	[108]

Abbreviations: DLBCL, diffuse large B-cell lymphoma; EEC, endometrioid endometrial carcinoma; MCL, mantle cell lymphoma; GCB, germinal B-cell–like.

In addition to PI3K pan-inhibition, several isoform-selective PI3K inhibitors have been shown to repress the viability of PTEN-deficient tumors. Notably, the p110β-specific PI3K inhibitor AZD6482 (KIN-193) displayed remarkable antitumor activity in PTEN-null tumors but failed to block the growth of PTEN–wild-type tumors in mouse models [98]. However, another separate study showed that endometrioid endometrial cancer with *PTEN* mutation were resistant to p110β-selective inhibition, cell lines' viability was decreased only when p110β-selective inhibition was combined with p110α-selective inhibition. Recent findings have highlighted that there is a complex interplay between the Class I PI3K isoforms, inhibition of either α or β single isoform might be compensated by reactivation of another isoform at last [99]. Furthermore, it has been proposed that the dual γ/δ inhibitor CAL-130, specifically targeting p110γ and p110δ isoforms in *PTEN* deleted T-ALL cell lines [100]. By contrast, Lonetti et al. recently indicated that PI3K pan-inhibition developed the highest cytotoxic effects when compared with both selective isoform inhibition and dual p110γ/δ inhibition, in T-ALL cell lines with or without *PTEN* deletion [101]. Nevertheless, which class of agents among isoform-specific or pan-inhibitors can achieve better efficacy is still controversial. Other target treatments including AKT, mTOR, dual PI3K/AKT and dual PI3K/mTOR inhibitors also show promising antitumor activity in cell line studies, and some of them have been testing under clinical trials [102–111] (Table 3, 4).

**Table 4:** Preclinical studies of targeted therapeutics in PTEN-deficient tumors

Inhibitor type	Drug	Patient population	Phase	Identifier
PI3K	GSK2636771	Patients with advanced solid tumors with PTEN deficiency	1/2a	NCT01458067
	BKM120	Patients with recurrent glioblastoma with PTEN mutations or homozygous deletion of PTEN or with PTEN-negative disease	1b/2	NCT01870726
	BKM120	Patients with advanced, metastatic, or recurrent endometrial cancers with PIK3CA gene mutation, PTEN gene mutation, or null/low PTEN protein expression	2	NCT01550380
	AZD8186	Patients with advanced CRPC, sqNSCLC, TNBC, or known PTEN-deficient advanced solid malignancies	1	NCT01884285
PI3K/mTOR	BEZ235	Patients with advanced TCC; group 1 includes patients with no PI3K pathway activation, no loss of PTEN, and no activating PIK3CA mutation; group 2 includes patients with PI3K pathway activation as defined by PIK3CA mutation and/or PTEN loss	2	NCT01856101
	BEZ235	Patients with relapsed lymphoma or multiple myeloma	1	NCT01742988
AKT	MK-2206	Patients with previously treated metastatic colorectal cancer enriched for PTEN loss and PIK3CA mutation	2	NCT01802320
	MK-2206	Patients with advanced breast cancer with a PIK3CA mutation, AKT mutation, and/or PTEN loss or mutation	2	NCT01277757
	Pazopanib + everolimus	Patients with PI3KCA mutations or PTEN loss and advanced solid tumors refractory to standard therapy	1	NCT01430572
	Trastuzumab +RAD001	Patients with HER-2–overexpressing, PTEN-deficient metastatic breast cancer progressing on trastuzumab-based therapy	1/2	NCT00317720
	GDC-0068/ GDC-0980 +abiraterone	Patients previously treated prostate cancer with PTEN loss (currently in phase II)	1b/2	NCT01485861
	Rapamycin (Temsirolimus)	Patients with advanced cancer and PI3K mutation and/or PTEN loss	1/2	NCT00877773
	Ipatasertib (GDC-0068) + paclitaxel	Patients with PTEN-low metastatic TNBC	2	NCT02162719

Abbreviations: CRPC, castrate-resistant prostate cancer; sqNSCLC, squamous non-small cell lung cancer; TNBC, triplenegative breast cancer; TCC, transitional cell carcinoma; HER-2, human epidermal growth factor receptor 2.

## CONCLUSION

In summary, recent studies have identified PTEN as a tumor suppressor gene in various human cancers. It is clear that PTEN is far more than a cytosolic protein that acts as a lipid phosphatase to maintain PIP_3_ levels. Therefore, we must reconsider the distinct roles PTEN have in specific subcellular compartments, identify the mechanisms underlying PTEN's shuttling between different compartments, and investigate the significance of these mechanisms in predicting disease outcome. Future studies will further elucidate the mechanistic basis of PTEN deficiency in lymphoid malignancies, thereby aiding in the clinical management of lymphoid malignancies with PTEN loss or alteration.
